# Rapid development of HIV elite control in a patient with acute infection

**DOI:** 10.1186/s12879-019-4374-8

**Published:** 2019-09-18

**Authors:** Deirdre Morley, John S. Lambert, Louise E. Hogan, Cillian De Gascun, Niamh Redmond, Rachel L. Rutishauser, Cassandra Thanh, Erica A. Gibson, Kristen Hobbs, Sonia Bakkour, Michael P. Busch, Jeremy Farrell, Padraig McGetrick, Timothy J. Henrich

**Affiliations:** 1 Mater Misericordae University Hospital, Eccles Street, Dublin, 7 Ireland; 2 Department of Medicine, University of California San Francisco Division of Experimental Medicine, 1001 Potrero Avenue, San Francisco, CA 94110 USA; 3 National Virus Reference Laboratory, University College Dublin, Dublin, Ireland; 4 UCD Clinical Research Centre, Dublin, Ireland; 5 Vitalant Research Institute, 270 Masonic Ave, San Francisco, CA 94118 USA; 6 University College Dublin School of Medicine, Dublin, Ireland; 7 Department of Laboratory Medicine, University of California, San Francisco, CA USA

**Keywords:** HIV-1, Elite control, Acute HIV infection, Antiretroviral therapy, HIV immune responses, HIV reservoirs

## Abstract

**Background:**

Elite controllers (EC), a small subset of the HIV-positive population (< 1%), suppress HIV viremia below the limit of quantification of clinical viral load assays in the absence of antiretroviral therapy (ART). However, there is a paucity of longitudinal data detailing the viral and immune dynamics or HIV reservoir seeding during acute infection in individuals that go on to become Elite Controllers.

**Case presentation:**

In this report, we describe a case of a 42 year old woman diagnosed during acute infection who rapidly and permanently suppressed her viremia in the absence of antiretroviral therapy (ART). Rapid antibody/antigen testing was either negative or equivocal during acute infection, despite subsequent viral load testing at that time point with 71,550 plasma HIV RNA copies/mL, making initial diagnosis challenging. The patient subsequently developed detectable anti-HIV antibodies and an increase in HIV-specific CD8+ T cell responses to overlapping subtype C HIV gag peptide; very low-level plasma viremia (0.84 RNA copies/mL) was detected by an ultrasensitive assay 2 years following infection. Subsequently, she was started on ART for multifocal furunculosis despite continued suppression of virus and stable CD4+ T cell counts. Following ART initiation, HIV specific antibody levels and CD8+ T cell responses increased, but no HIV DNA or RNA was able to be isolated from large numbers of peripheral blood CD4+ T cells.

**Conclusion:**

This case provides important information regarding the establishment of elite HIV control during acute infection and also demonstrates an increase in HIV-specific immune responses following ART despite undetectable peripheral blood cellular measures of HIV persistence. This case also highlights the challenges in diagnosing acute HIV infection without the use of viral load testing in this rare elite controller phenotype.

## Background

Acute HIV-1 infection is characterized virologically by a high viral load, followed by the establishing of a lower viral load *set point* [1, 2]. Following acute infection, the natural evolution of HIV is defined by persistent depletion of peripheral CD4 T cells, immune amplification, and inflammation [1, 3]. Elite controllers, a small subset of the HIV-positive population (< 1%), suppress HIV viraemia below the limit of quantification of clinical viral load assays in the absence of ART [1, 3–6]. Elite control is, to a large part, a result of cellular host genetic and immune responses, and has been a model for immune-based studies to achieve long-term ART-free remission. However, there is a paucity of data detailing the viral and immune dynamics during acute infection in individuals that go on to become ECs.

We identified an individual who presented in the early Fiebig stage 1 of acute HIV infection (detectable plasma HIV RNA prior to detectable viral antigenemia or HIV-specific antibody responses) and subsequent rapid spontaneous decay of plasma viremia leading to diagnostic and therapeutic challenges. Since it is very rare to have longitudinal data and samples available from the time of acute infection through development of control [7], this case provided the opportunity to study evolving HIV-specific adaptive and humoral responses and allowed in depth characterization of cell-associated HIV-1 RNA, DNA and low-level residual viremia by ultrasensitive assays. In addition, we had the opportunity to study changes in HIV reservoirs and immune responses prior to and following initiation of ART, which was based on clinical manifestations rather than increasing HIV burden or CD4+ T cell decline.

## Case presentation

An asymptomatic 42-year old woman presented to our sexual health clinic 1 month following unprotected sexual intercourse with a high risk male partner whom was later presumed to be the source of infection. She denied any other high risk sexual contacts at the time. A HIV Antigen/Antibody (Ag/Ab) test was reported as negative at that time, but she represented 5 months later for a repeat screen at which time a 4th generation Ag/Ab test was reactive. HIV line immunoassay testing was consistent with recent HIV-1 acquisition*,* and plasma HIV RNA at this time was detectable but below the level of quantification (< 200 copies/ml).The individual continued to follow up for routine clinical care, and plasma HIV RNA became undetectable 4 months following her initial positive test as shown in Table 1. Her CD4 count remained stable, but 2 years following diagnosis, she presented with groin and axillary furunculosis. She was treated with antibiotics however her skin condition did not improve to a satisfactory degree. A decision was made to commence abacavir/lamivudine/dolutegravir ART on clinical grounds. This subsequently improved with ART administration.

**Table 1:** Results of HIV molecular testing, Antigen/Antibody screening assay, and CD4 count from time first presentation (June 2014) to initiation of ARV therapy

Time	Viral Load (RNA copies/ml)		Forth Generation Antigen/Antibody HIV Test (s/co –relative quantity of HIV Ab)				Confirmatory Test	CD4+ T Cells/uL (%)
	Viral Load	SCA	ARCHITECT (S/Co)	VIDAS (S/Co)	GS		INNO-LIA	
Jun 2014	71550^a^	–	*1* ^b^	NEG	NEG	*–*	–	–
Oct 2014	< 200	–	11.1^c^	13.72^c^	–	–	gp41 (3+), p31 (1+), p24 (3+), p17 (1+)^c^	–
Nov 2014	< 40	–	–	–	–	–	–	616 (45%)
Dec 2014	–	–	–	–	–	–	–	–
Oct 2015	< 40	–	–	–	–	–	–	459 (46%)
Apr 2016	< 40	–	–	–	–	–	–	558 (40%)
Sep 2016	–	0.84^d^	–	–	–	13^c^	–	–
Antiretroviral therapy commenced September 2016^e^								
Oct 16	–	–	–	–	–	70.8^c^	–	585 (47%)

*SCA* single copy assay, *S/Co* signal/cutoff

Fourth Generation HIV Antigen/Antibody test: ARCHITECT® Abbott; VIDAS® BioMerieux; *GS = GeneScreen*® Bio-Rad, VITROS® Ortho Clinical diagnostics (Ab level quantified)

*INNO-LIA, Fujirebio*®

^a^Retrospective molecular test on stored sample from June 2014

^b^Read as equivocal value at time of testing

^c^Positive test

^d^Sample was taken prior to ARV commencement in Sept 2016

^e^ARV commenced on clinical grounds-patient presented with furunculosis

Subsequent review of the sample taken at her initial STI screen revealed that the first-line 4th generation Ag/Ab (ARCHITECT® Abbott) screening assay result was at the threshold of positivity. However, this result was not confirmed on two other 4th generation tests. The clinical suspicion was low at the time, as her partner was not known to be high risk, and no further testing was performed. Retrospective HIV RNA testing of this sample, however, revealed a viral load of 71,550 copies/ml (Table 1). Genotypic testing confirmed the presence of a Group M, subtype C virus. HLA typing demonstrated B27 positivity, which has been reported to be enriched in HIV controllers [8].

Informed consent was obtained and the patient provided blood for further analysis. Single copy viral load testing of a sample from September 2016 (acquired 27 months following infection and prior to initiation of ART) was performed using replicate Aptima viral load Target-Capture Transcription-Mediated-Amplification (TC-TMA) assay on the Panther system (Hologic), which detected 0.84 plasma copies/ml (3 positive replicates of 13 performed). Purified peripheral blood CD4+ T cells were tested from six samples obtained longitudinally between December 2015 and January 2017 (before and after ART initiation) for total cell-associated HIV-1 DNA and unspliced RNA testing using previously described quantitative PCR methods (10 to 20 million input cells per experiment) [9, 10]. Exogenous activation for 48 h using αCD3/αCD28 antibodies was incorporated to increase detection of HIV-1 RNA from cells. No cell-associated HIV-1 DNA or RNA was detected in any of these samples.

**Fig. 1:**
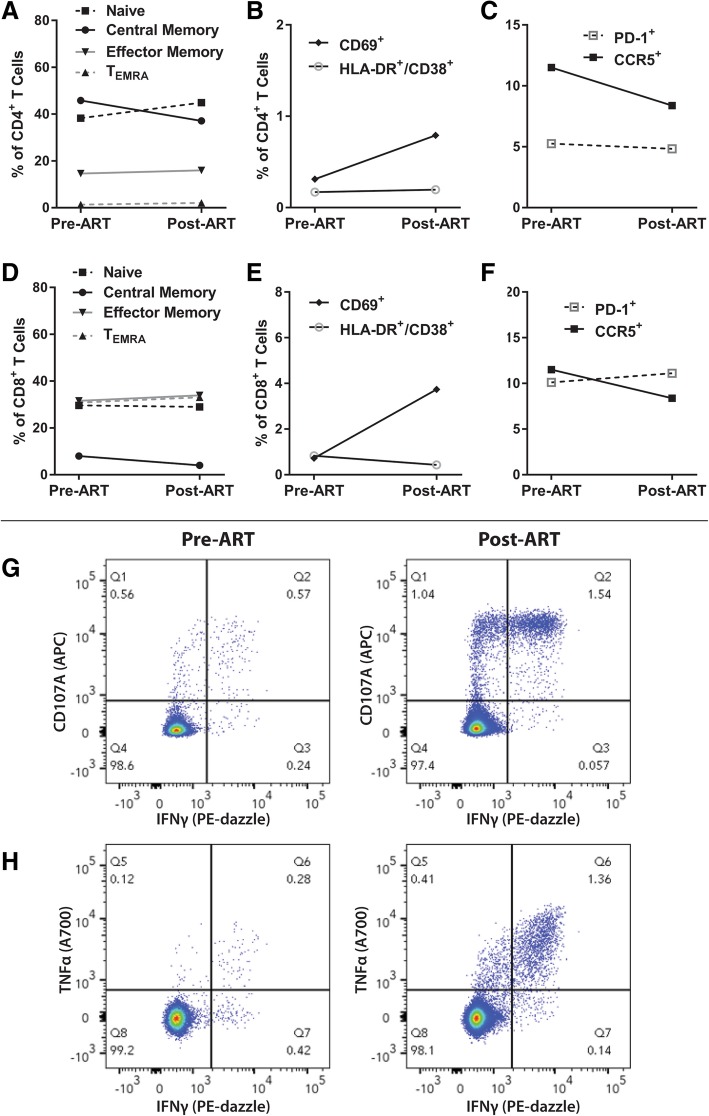
Immune phenotypes and HIV-specific T cell responses prior to and following ART imitation in the setting of EC. CD4+ and CD8+ T cell immune phenotyping prior to and following initiation of antiretroviral therapy. Changes in CD4+ T cell subsets, markers of early and late activation, and PD-1 and CCR5 expression are shown in **a**-**c**. Changes in frequencies of CD8+ T cells expressing subset, activation, immune checkpoint, and CCR5 are shown in **d**-**f**. Frequencies of cells co-expressing CD107a and Interferon (IFN) γ (**g**) and Tumor Necrosis Factor (TNF) α and Interferon (IFN) γ (**h**) are shown prior to and following initiation of ART. Results shown in all panels are from sampling at least 2 years following acute HIV infection

Flow cytometry was performed to determine the frequency of markers of CD4+ and CD8+ T cell subset differentiation (CD45RA; CCR7), activation (CD69; HLA-DR/CD38), immune checkpoint (PD-1) and CCR5 expression prior to and following initiation of ART (September 2016 and November 2016). No major changes in surface marker expression were observed before and after ART initiation with the exception of modest increases in the frequency of CD8+ T cells expressing CD69, and decreases in CCR5 expression on both CD4+ and CD8+ T cells (Fig. 1a-f). In addition, HIV-specific CD8+ T cell responses were measured in samples obtained in June 2016 and following initiation of ART in January, 2017 by cell surface and intracellular staining as in supplementary materials following 6 h stimulations using HIV subtype C overlapping, pooled Gag peptides (obtained from the NIH AIDS Reagent Repository). The frequency of CD107a + (a toxic degranulation marker) and intracellular TNFα+ and IFNγ+ expressing CD8+ T cells increased following initiation of ART as shown in Fig. 1g, h.

## Discussion and conclusions

Elite controllers, a small subset of the HIV-positive population (< 1%), suppress HIV viremia below 50 copies per ml in the absence of ART [4]. However, there is a paucity of data detailing the viral and immune dynamics during acute infection in individuals that go on to become ECs. Recently, two cases have been reported involving women with subtype C infection that developed elite control and were followed longitudinally from early infection to development of control [7]. Like our case, these individuals also had HLA haplotypes previously associated with slow disease progression, but one participant developed elite control in 6 weeks whereas the other took 6 months [7]. The prior participant with rapid control developed early and robust HIV-specific CD8+ T cell responses, but these waned years after initial infection. Interestingly, the second participant had pre-existing CD8+ T cell responses [7]. Our case also developed high frequencies of HIV-specific CD8+ T cell responses, but unlike in the prior study, these responses continued to increased years after initial infection and after the initiation of ART for non virologic reasons. The frequency of CD8+ T cell responses can be variable in ECs, and given limited sampling, the increase may be simply due to temporal fluctuations in responses. Unfortunately, we were unable to obtain tissue for this study, and more in-depth sampling may have provided further insight into HIV-specific responses. Regardless, CD + T cell responses were high overall throughout the study and may explain, in part, the levels of extraordinary control observed in this individual.

This case provides important information regarding the establishment of HIV control during acute infection which was characterized by a relatively high initial viral load at the time of initial Ab seroconversion, as would be expected in a majority of acute infections. The peak viremia of 71,550 copies/mL measured in our participant was higher than the peak viral loads observed in the prior two reported cases (all values were less than 13,000 copies/mL). Peak viremia was followed by a relatively rapid loss in detectable viral load measurements by commercial assays. The decrease in viral load occurred around the time when non-controllers start to achieve a viral set point as a result of partial immune control of viral replication [11].

This case is also unique in the spontaneous restriction of reservoir seeding by immune control as demonstrated by a lack of nucleic acid detection in tens of millions of peripheral CD4+ T cells, despite exogenous, in vitro activation and quantitative PCR testing of samples using a highly sensitive assay with near single-copy sensitivity across HIV subtypes as previously described [9, 12]. Given lack of detection of HIV DNA or RNA following exogenous activation of tens of millions of CD4+ T cells, quantitative outgrowth or sequencing to look for intact or replication competent virus was not possible. HIV reservoir data were not reported for the prior cases discussed above [7]. A majority of ECs have detectable HIV DNA and RNA in cells and tissues despite low plasma RNA levels [13], with the exception of the very rare phenotype of the “extraordinary” controller [6]. Plasma RNA testing using ultrasensitive quantitation methods revealed the persistence of very low-level, residual plasma HIV RNA prior to ART initiation. T cell activation can also be persistently elevated in EC, and may predict progression of HIV-related disease and need for ART [13–16]. Overall levels of activation in our participant’s samples were low prior and following initiation of ART in this individual, which may be explained by very low HIV burden following development of viral control.

Individuals presenting in the first 2 weeks following infection prior to the development of an antibody response are at risk of their diagnosis being missed unless viral load testing is performed [17]. Following this period, however, combined fourth generation HIV Ag/Ab assays detect seroconversion either through the identification of HIV-1 Gag p24 protein antigen, or through the presence of HIV-specific antibodies [1, 3, 18]. These assays are sensitive and specific and usually able to detect antigen if HIV RNA is > 30,000 copies/ml [1, 19]. Nonetheless, this patient’s diagnosis could have been made earlier with viral load testing, as she presented 1 month following what was subsequently identified as a high-risk sexual encounter. It is therefore important to review test results in conjunction with the level of clinical suspicion and the pre-test probability.

## Data Availability

Data is available as required. The corresponding author Deirdre Morley should be contacted.

## Abbreviations

Ab: Antibody
Ag: Antigen
ART: Antiretroviral Therapy
EC: Elite Controller
HIV: Human Immunodeficiency Virus
RNA: Ribonucleic acid
