# Correction to: Rapid development of HIV elite control in a patient with acute infection

**DOI:** 10.1186/s12879-019-4485-2

**Published:** 2019-10-22

**Authors:** Deirdre Morley, John S. Lambert, Louise E. Hogan, Cillian De Gascun, Niamh Redmond, Rachel L. Rutishauser, Cassandra Thanh, Erica A. Gibson, Kristen Hobbs, Sonia Bakkour, Michael P. Busch, Jeremy Farrell, Padraig McGetrick, Timothy J. Henrich

**Affiliations:** 1 Mater Misericordae University Hospital, Eccles Street, Dublin 7, Ireland; 2 Department of Medicine, University of California San Francisco Division of Experimental Medicine, 1001 Potrero Avenue, San Francisco, CA 94110 USA; 3 National Virus Reference Laboratory, University College Dublin, Dublin, Ireland; 4 UCD Clinical Research Centre, Dublin, Ireland; 5 Vitalant Research Institute, 270 Masonic Ave, San Francisco, CA 94118 USA; 6 University College Dublin School of Medicine, Dublin, Ireland; 7 Department of Laboratory Medicine, University of California, San Francisco, CA USA

**Correction to: BMC Infect Dis**

**https://doi.org/10.1186/s12879-019-4374-8**

After publication of the original article [1], we were notified that a column needed to be removed from Table 1.

The correct version can be found below:

**Table 1:** Results of HIV molecular testing, Antigen/Antibody screening assay, and CD4 count from time first presentation (June 2014) to initiation of ARV therapy

Time	Viral Load (RNA copies/ml)		Forth Generation Antigen/Antibody HIV Test (s/co –relative quantity of HIV Ab)			Confirmatory Test	CD4+ T Cells/uL (%)
	Viral Load	SCA	ARCHITECT (S/Co)	VIDAS (S/Co)	GS	INNO-LIA	
Jun 2014	71550^a^	–	*1* ^*b*^	NEG	NEG	–	–
Oct 2014	< 200	–	11.1^c^	13.72 ^c^	–	gp41 (3+), p31 (1+), p24 (3+), p17 (1+) ^c^	–
Nov 2014	< 40	–	–	–	–	–	616 (45%)
Dec 2014	–	–	–	–	–	–	–
Oct 2015	< 40	–	–	–	–	–	459 (46%)
Apr 2016	< 40	–	–	–	–	–	558 (40%)
Sep 2016	–	0.84^d^	–	–	–	–	–
Antiretroviral therapy commenced September 2016 ^d^							
Oct 16	–	–	–	–	–	–	585 (47%)

Fourth Generation HIV Antigen/Antibody test: ARCHITECT® Abbott; VIDAS® BioMerieux; *GS = GeneScreen*® Bio-Rad, *INNO-LIA, Fujirebio*®

*SCA* Single copy assay, *S/Co* Signal/cutoff

^a^ Retrospective molecular test on stored sample from June 2014

^b^ Read as equivocal value at time of testing

^c^ Positive test

^d^ ARV commenced on clinical grounds-patient presented with furunculosis

Furthermore, the words “Hiv specific antibody levels and” should be deleted from the ‘Case presentation’ section in the Abstract.

The original article has been corrected.

The publisher apologies for the inconvenience.
